# Application of *in vitro* bioaccessibility and bioavailability methods for calcium, carotenoids, folate, iron, magnesium, polyphenols, zinc, and vitamins B_6_, B_12_, D, and E

**DOI:** 10.3389/fphys.2012.00317

**Published:** 2012-08-06

**Authors:** Paz Etcheverry, Michael A. Grusak, Lisa E. Fleige

**Affiliations:** ^1^Department of Pediatrics, USDA-ARS Children's Nutrition Research Center, Baylor College of Medicine, HoustonTX, USA; ^2^Global Research and Development, PepsiCo, BarringtonIL, USA

**Keywords:** *in vitro* methods, minerals, vitamins, bioaccessibility, bioavailability

## Abstract

A review of *in vitro* bioaccessibility and bioavailability methods for polyphenols and selected nutrients is presented. The review focuses on *in vitro* solubility, dialyzability, the dynamic gastrointestinal model (TIM)™, and Caco-2 cell models, the latter primarily for uptake and transport, and a discussion of how these methods have been applied to generate data for a range of nutrients, carotenoids, and polyphenols. Recommendations are given regarding which methods are most justified for answering bioaccessibility or bioavailability related questions for specific nutrients. The need for more validation studies in which *in vivo* results are compared to *in vitro* results is also discussed.

## Introduction

Throughout the years, *in vitro* screening methods have been developed and refined for the determination of nutrient bioaccessibility and bioavailability from foods. These are methods that can provide useful information, especially when one considers the vast number of factors that can affect nutrient absorption. Bioavailability, which is defined as the amount of an ingested nutrient that is absorbed and available for physiological functions, is dependent on digestion, release from the food matrix, absorption by intestinal cells, and transport to body cells. Bioaccessibility, which is the amount of an ingested nutrient that is potentially available for absorption, is dependent only on digestion and release from the food matrix.

It has to be kept in mind that bioavailability, which has a physiological or metabolic endpoint, can never be measured in its entirety by any of these *in vitro* methods. Furthermore, host factors that can possibly influence nutrient absorption such as nutrient status, age, genotype, physiological state (e.g., pregnancy, lactation, and obesity), chronic and acute infectious disease states, secretion of hydrochloric acid, gastric acid, and/or intrinsic factor, are impossible to factor in *in vitro* assays. Nonetheless, for this review, we will use the term bioavailability in order to retain the terminology used by many of the authors referenced here. However, we urge readers to be cautious when interpreting *in vitro* “bioavailability” data, and that they verify which aspect of the bioavailability process is being assessed. In many cases, researchers are only measuring uptake or absorption with their *in vitro* method, yet refer to their analysis as bioavailability.

*In vitro* bioaccessibility/bioavailability methods are useful to provide knowledge on possible interactions between nutrients and/or food components, the effects of luminal factors (including pH and enzymes), food preparation and processing practices, nature of the food matrix etc., on either micronutrient absorbability (a component of bioavailability) or on the potential for a nutrient to be absorbed (i.e., bioaccessibility). *In vitro* methods are less expensive, faster, and offer better controls of experimental variables than human or animal studies (Sandberg, 2005). However, *in vitro* studies cannot be substituted for *in vivo* studies, and should be therefore regarded as a screening, ranking, or categorizing tool.

## *In vitro* methods

There are principally four *in vitro* methods for measuring bioaccessibility and/or bioavailability: solubility, dialyzability, or a gastrointestinal model (e.g., TIM) for bioaccessibility, and the Caco-2 models for bioavailability (Table 1).

**Table 1:** ***In vitro* screening methods**.

***In vitro* method**	**End point**	**Advantages**	**Limitations**
**Solubility**	Measures bioaccessibility	Simple to do Relatively inexpensive Easy to conduct, every laboratory would have the necessary equipment	Sometimes not a reliable indicator of bioavailability Cannot assess rate of uptake or absorption or transport kinetics Cannot measure nutrient or food component competition at the site of absorption
**Dialyzability**	Measures bioaccessibility	Simple to do Relatively inexpensive Easy to conduct, every laboratory would have the necessary equipment	Cannot assess rate of uptake or absorption or transport kinetics Cannot measure nutrient or food component competition at the site of absorption
**Gastrointestinal models**	Measures bioaccessibility. However, when coupled to intestinal cells, bioavailability can also be measured	Incorporates many digestion parameters (peristalsis, churning, body temperature, etc.,) Allows the collection of digest at any step of the digestive system	Expensive Few validation studies
**Caco-2 cell model**	Measures bioavailability	• Allows the study of nutrient or food component competition at the site of absorption	• Requires trained personnel with knowledge of cell culture methods

In each of these methods, an *in vitro* digestion is conducted to simulate the human digestive system via a two-step (sometimes a three-step) digestion that includes a gastric and intestinal digestion. For the gastric digestion, pepsin (from porcine stomach) is added prior to the acidification of the samples to pH 2 (to simulate the gastric pH of an adult) or to pH 4 (to simulate the gastric pH of an infant). Acidification of the samples to pH 2 or 4 is important, because pepsin begins to denature itself and thus will lose its activity at pH ≥ 5. Before the start of the intestinal digestion, the samples are neutralized to pH 5.5–6 prior to the addition of pancreatin (which consists of a cocktail of pancreatic enzymes such as pancreatic amylase, lipase, ribonuclease, and proteases such as trypsin) and bile salts (which are emulsifiers), and finally re-adjusted to pH 6.5–7. The third digestion step that is sometimes introduced, and which precedes the gastric phase, is the digestion by lingual alpha-amylase, which is an enzyme that breaks apart the glycosidic bonds of starch molecules, i.e., amylose and amylopectin. Once the food in question has been digested, bioaccessibility can either be measured via solubility, dialyzability or gastrointestinal models.

For the solubility assay, the intestinal digests need to be centrifuged, to yield a supernatant and precipitate. The nutrients or compounds present in the supernatant represent the soluble components and are measured by atomic absorption spectrophotometry (AAS), mass spectrometry, spectrophotometry, inductively coupled plasma atomic emission spectroscopy (ICP-AES), high performance liquid chromatography (HPLC), or in the case of radioactive compounds, by gamma or liquid scintillation counting. Percent solubility is calculated as the amount of soluble compound relative to the total amount of compound in the test sample.

Dialyzability assays were introduced in 1981 by Miller et al. as a means to estimate iron bioaccessibility from foods. The model, which measures soluble minerals of low molecular weight, is based on an equilibrium dialysis. It involves the addition of a dialysis tubing of a certain molecular weight cut off (MWCO), following the gastric digestion. The dialysis tubing or bag contains a buffer, such as sodium bicarbonate, that slowly diffuses out of the bag and neutralizes the peptic digest. After incubation, pancreatin/bile is added and following another incubation total dialyzable iron can thus be determined by measuring the amount of mineral present in the dialysate. The whole premise of dialyzability methods is that dialyzable compounds will be available for absorption in the small intestine. This method has been applied and slightly modified to study the bioaccessibility of a number of micronutrients including calcium, zinc, and magnesium, among others. An extension to this method involves the continuous-flow dialysis system performed by means of a hollow-fibre system (Wolters et al., 1993). As opposed to the *in vitro* methods based on Miller et al. (1981), in which components that pass the dialysis membrane are not removed, the continuous-flow dialysis system takes the removal of dialysable components into account leading probably to a better estimate of *in vivo* bioavailability.

A number of institutions and commercial groups have developed sophisticated gut models to simulate the human digestive system (Afkhami et al., 2007; de Jong et al., 2007; Barmpalia-Davis et al., 2008; van den Abbeele et al., 2010; Vardakou et al., 2011). One commercial gastrointestinal model (TIM), which has been developed by The Netherlands Organization (TNO) for Applied Scientific Research, has been described in great detail by Minekus et al. (1995, 1999). TNO's intestinal model (TIM) is a very sophisticated model since many parameters of the human digestive system are simulated: e.g., body temperature, flow of saliva, gastric- and pancreatic juice including digestive enzymes, and bile, peristalsis and churning, gastrointestinal transit times, regulation of gastric and intestinal pH, etc. The model consists of two computer-controlled chambers, named TIM1 and TIM2. TIM1 comprises four compartments that represent the stomach, duodenum, jejunum, and ileum. Secretion of digestive juices and pH adjustment in each section are simulated according to physiological data. A dialysate component collects compounds and they represent the bioaccessible fraction. The material that exits the model represents, on the other hand, the nonbioaccessible fraction and is used to study colonic fermentation products in the TIM2 (Anson et al., 2009). TIM2 represents the human large intestine, where the colonic fermentation experiments are performed. The nonbioaccessible fraction generated from TIM1 can be inoculated with active microbes obtained from humans. One of the main advantages of the TIM system is the possibility of collecting samples at any level of the gastrointestinal tract and at any time during digestion (Etienne-Mesmin et al., 2011). Although this model measures bioaccessibility, bioavailability can also be measured if the food digest at the end of the TIM1 digestion is added to human intestinal cells and nutrient uptake is assessed (TNO, 2011).

Bioavailability (or more correctly, components of bioavailability) can be assessed through the determination of nutrient uptake, transport, or both by Caco-2 cells. Caco-2 cells belong to a human epithelial cell line derived from a human colonic adenocarcinoma. Even though they have a colonic origin, for reasons that to this day are not understood, the cells behave very much like intestinal cells upon culture. Uptake studies are performed with cells grown on the surface of plastic dishes or wells, or alternatively, if transport will also be measured, on Transwell inserts. Transwell inserts allow the collection and measurement of nutrients that have been absorbed through the apical membrane and then released through the basolateral membrane. Following the gastric digestion of the food, pancreatin/bile is added and the digest is added to the cells. *In vivo*, cellular integrity is maintained through the presence of an intestinal mucus layer. However, *in vitro*, one of several methods must be used to prevent the enzymatic degradation of the cells. One method is the introduction of a dialysis membrane secured with a silicone O-ring to a plastic insert, which is placed on top of the cell monolayer. The intestinal digest is placed on top of the dialysis membrane, thus preventing the enzymes from reaching the cells (Gangloff et al., 1996; Glahn et al., 1998). Another method involves heat treating the intestinal digests for 4 min at 100°C in order to inhibit the enzymes added during the experiment (Jovaní et al., 2001; Frontela et al., 2009). This step, however, imposes a shortcoming in the methodology, because heating the sample at 100°C will also likely denature food proteins, thus impacting (either positively or negatively) bioavailability. Other methods involve the inactivation of the enzymes by acidifying the intestinal digests to pH 2 (Frontela-Saseta et al., 2011) or by lowering the temperature of the digests and subsequently filtering the samples (Au and Reddy, 2000). However, these steps are not physiologically representative of *in vivo* conditions. The *in vitro* co-culture of Caco-2 and HT29-MTX, a human mucus-producing cell line, might represent a more physiological and realistic approach to *in vivo* conditions (Mahler et al., 2009), as the generated mucus layer would protect the Caco-2 cells from digestive enzymes. This approach has not been used extensively; thus, more studies are needed to determine its general applicability for various nutrients, and to evaluate the consequences of incorporating an additional diffusional layer to the apical membrane of the Caco-2 cells.

The Caco-2 uptake of some, but not all, dietary micronutrients has been examined. In the case of carotenoids and other fat soluble compounds, it is the Caco-2 uptake of either micellarized or soluble (but not necessarily micellarized) compounds that is assessed. Iron uptake can be estimated via ferritin formation or ^59^Fe uptake (a radioisotope which had been allowed to equilibrate with the food in question). Unlike ferritin formation, which is an indicator of iron uptake, there are no biomarkers of uptake for minerals like calcium and zinc. The use of metallothionein, a cytoplasmic protein that stores zinc, as an indicator of zinc uptake has some potential. However, metallothionein can also bind and store other metals like copper, selenium, cadmium, mercury, silver, and arsenic (Bell and Vallee, 2009). Thus, the protein is not specific for zinc which questions the suitability of this biomarker for measuring zinc bioavailability. Cellular calcium and zinc uptake have been determined by measuring cell uptake via atomic absorption spectroscopy. However, in this method one cannot differentiate the calcium or zinc originally present in the cells from the minerals that have been absorbed from the digested food, since one is measuring total mineral content. Alternatively, radioisotopic forms of the minerals can be used and traced. However, this has certain complications that have to be addressed such as radioactivity exposure, appropriate rinse solution to remove surface bound radioisotopes, increased costs, and the possible lack of an equilibration between the isotope and the endogenous mineral present in the food, among others.

Caco-2 transport studies require that the cells grow on Transwell inserts containing semipermeable membranes, thus allowing the formation of two chambers: an apical chamber which receives the digested test meal and a basolateral chamber where the transported compound can be collected and later analyzed. Cell monolayer integrity on Transwell inserts has to be monitored and most often is done by measuring transepithelial electrical resistance (TEER) across the cell monolayer or by measuring the amount of a nontransportable fluorescent compound such as luciferase yellow. An optimal monolayer integrity test result suggests that tight junctions between adjacent epithelial cells exist, thus providing a good separation between the apical and the basolateral chambers.

## Applications of *In vitro* methods and recommendations

### Calcium

Calcium is a macromineral that plays an important role in bone health, muscle contraction, blood clotting, nerve conduction, enzyme regulation, and possibly weight loss (Guéguen and Pointillart, 2000; Tremblay and Gilbert, 2011). In humans, intestinal calcium absorption is controlled by complex homeostatic mechanisms involving calcitriol and the parathyroid hormone (PTH). Calcitriol (1,25(OH_2_) vitamin D_3_) increases the synthesis of a cytosolic calcium-binding protein (calbindin) resulting in increased calcium transport in intestinal cells (DeLuca, 1985). The PTH indirectly affects intestinal calcium absorption by increasing the formation of calcitriol from its precursor, calcidiol (25(OH) vitamin D_3_) (Raisz, 1981). This internal regulation of intestinal absorption certainly makes it difficult to rely on *in vitro* availability results as an estimation of calcium bioavailability.

However, regardless of the mechanism involved in calcium homeostasis, calcium has to be soluble in the gastrointestinal tract before it can be absorbed. Certain dietary factors can impact calcium solubility, thereby affecting calcium bioavailability at the absorptive surface of intestinal cells. Thus, *in vitro* methods might be useful to compare the bioaccessibility/bioavailability of different calcium salts that are contained in dietary supplements or when added as food fortificants (e.g., calcium carbonate, calcium citrate, calcium phosphate, calcium gluconate, etc.). These methods can also be used to assess the effects of the type of protein present in foods, the effect of digestible carbohydrates such as lactose, and non-digestible carbohydrates such as fibers and carbohydrates gums, or plant food components including phytate, and fructo-oligosaccharides on calcium bioaccessibility/bioavailability (Cámara-Martos and Amaro-López, 2002). Furthermore, calcium has the tendency to bind to fatty acids in the lumen forming insoluble soaps. Thus, studying which types of fatty acids (i.e., short vs. long chain, saturated vs. unsaturated) lead to a more absorbable form of the mineral will be very easy to conduct in an *in vitro* type of experiment. Below are some of the dietary factors affecting calcium bioavailability, which have been studied via *in vitro* methods.

#### Casein phosphopeptides

Casein phosphopeptides (CPPs) result from the enzymatic hydrolysis of casein, the predominant protein found in cow's milk. CPPs contain clusters of phosphoserine residues, which can effectively bind calcium, and inhibit formation of insoluble calcium phosphates (Narva et al., 2003). Pure CPPs have been shown to promote calcium absorption in *in vitro* assays using HT-29 cells and Caco-2 cells (Ferraretto et al., 2003; Cosentino et al., 2010) and *in vivo*. Erba et al. (2001) who studied the intestinal calcium absorption in rats found that the absorption from CaCl_2_ solutions decreased by 90% when in the presence of phosphate (Ca:Pi molar ratio of 1:1), but decreased by only 40% from Ca-CPP at the same Ca:Pi molar ratio.

On the other hand, Drago and Valencia (2004) who used an *in vitro* dialyzability assay to measure bioaccessibility from infant formulas found no increase in calcium dialyzability with increasing casein concentration, perhaps due to an incomplete casein proteolysis. Kennefick and Cashman (2000) similarly found no effect of three different casein phosphopeptide preparations on calcium dialyzability. A human study performed on nine Finnish postmenopausal women who received milk and milk enriched with CPPs, found no differences in serum calcium between the two groups. According to the authors, a stimulatory effect of CPPs on calcium absorption might have been observed had the subjects been vitamin-D deficient (Narva et al., 2003). Likewise, a calcium lactate drink supplemented with CPPs led to *lower* fractional absorption of calcium in adults than the unsupplemented kind (*P* = 0.015). Thus, there appears to be conflicting results on the effects of CPPs both in *in vivo* and in *in vitro* experiments, and more experiments are needed to clarify their role in mineral bioavailability.

#### Phytate

Components in plant foods like phytate can form insoluble complexes with calcium, thereby reducing its bioavailability. Kennefick and Cashman (2000) reported that phytate had a more pronounced negative effect on calcium solubility than oxalate, wheat fibre-extract, barley fibre-extract, and casein. Liang et al. (2010), who used an *in vitro* solubility assay to compare rice-based foods from China, found that the high level of phytate in the brown rice (ranging from 14.9 to 19.4 mg of phytic acid/gram of rice) resulted in the lowest calcium solubility (12%) among all the rice foods tested. Brown rice germination, a process that results in phytate hydrolysis (Schlemmer et al., 2009), increased calcium solubility from 12% to 18%. Not surprisingly, the calcium solubility of white rice, which was produced by milling and polishing of the brown rice to remove the outer layer, increased with respect to brown rice (16.2% vs. 12%). Rice noodles, which are soaked and fermented prior to noodle making, had a percent calcium solubility ranging from 33.7% to 38.2% probably as a result of the low levels of phytic acid present (ranging from 0.0 to 4.1 mg of phytic acid/gram of rice noodles).

Phytate's inhibitory role on calcium absorption is significant only when the phytate to calcium molar ratio is above a certain value; below that value, the inhibitory effect is trivial. According to Frontela et al. (2009), the cut-off value is a molar ratio of 0.24. The authors, who used an *in vitro* digestion/Caco-2 cell uptake model to compare three different commercial cereals sold in Spain, found that calcium uptake was higher from infant cereals which had been dephytinized. However, results were significant (*P* < 0.05) only for the infant cereal which contained the highest phytate to calcium molar ratio. The other infant cereals tested had a phytate to calcium molar ratio ≤ 0.18 (Frontela et al., 2009).

Using dialyzability assays, Kamchan et al. (2004) found that vegetables containing the highest *in vitro* dialyzability for calcium (20–39%) corresponded to the ones that contained the lowest levels of phytate, fiber, and oxalate (e.g., kale, celery, collard, Chinese cabbage, and soybean sprouts). On the other hand, low dialyzable calcium (2–7%) corresponded to samples with high levels of oxalate and phytate (e.g., amaranth, white, and black sesame seeds).

#### Carbohydrates

Soluble fibers may have negative or positive effects on calcium absorption. In some European countries, carbohydrate gums such as alginic acid, guar gum, and locust bean gum are used as thickeners in commercial anti-regurgitation milk formulas for infants with evidence of gastroesophageal reflux (Bass and Chan, 2006). Bosscher et al. (2000) found that the incorporation of locust bean gum into an anti-regurgitation infant formula significantly lowered calcium dialyzability (9.4% ± 0.7%; *P* < 0.01) in comparison with the corresponding nonthickened formula (13.3% ± 1.2%). According to the authors, locust bean gum appears to affect calcium dialyzability by means of its physical properties to act as a thickening agent, rather than to its chemical ability to form complexes (Bosscher et al., 2003a). In another *in vitro* study, calcium availability was similarly reduced after supplementation with locust bean gum (11.9%) and high esterified pectin (11.7%), but it increased by 30% after inulin supplementation (Bosscher et al., 2003b). The ability of inulin to enhance calcium absorption has also been shown both in human (Abrams et al., 2005, 2007; Holloway et al., 2007) and animal (Coudray et al., 2005; Raschka and Daniel, 2005) studies.

#### Maillard reaction products and other processing conditions

Maillard reaction products are compounds in foods or beverages that are generated in the presence of heat, amino acids, and reducing sugars. The Maillard reaction induces browning of foods, has an effect on nutritive value, can have toxicological implications (such as the formation of acrylamide), can produce antioxidative components and it has also a large effect on flavor (van Boekel, 2006). Furthermore, Maillard reaction products may affect calcium bioavailability. Seiquer et al. (2010) used an *in vitro* digestion/solubility assay to compare the effect on calcium of thermally damaged milk, by comparing overheated milk (three cycles of sterilization at 116°C, 16 min) with ultra-high temperature (UHT) milk (150°C, 6 s). Calcium solubility was lower from the overheated milk, which has higher concentrations of Maillard reaction products, than from the UHT milk. The results were validated against rat feeding trials. Feeding rats the diet containing the overheated milk as the main protein source led to significantly lower values of apparent calcium absorption and retention than those found among animals fed the UHT milk diet. On the other hand, Mesías et al. (2009) found no effect of Maillard reaction products on Caco-2 calcium transport. The authors used two diets: a “white diet (WD)” (low in Maillard reaction products) and a “brown diet (BD)” (high in Maillard reaction products). For the preparation of the WD, cooking practices in which the Maillard reaction products develop (i.e., frying, toasting, and roasting) were avoided. The BD was rich in processed foods (breakfast cereals, baked products, chocolate, fried foods, toasted foods, and breaded foods, etc.,) with an evident development of browning and, thus, rich in Maillard reaction products. When 20 male adolescents were fed the two diets using a randomized crossover trial, there were also no differences in bioavailability (% calcium absorption; WD = 40.4%, BD = 38.2%) (Mesías et al., 2009).

Processing conditions were also tested. Viadel et al. (2006) used the Caco-2 cell uptake model to assess the effect of cooking on calcium availability. The bioavailability of calcium from cooked white beans (*Phaseolus vulgaris* L.) was higher (calcium uptake 18.8%) than from the raw beans (3.6%). Repo-Carrasco-Valencia et al. (2010) showed that boiled kañiwa (*Chenopodium pallidicaule*), a grain that grows in the Andes, had higher calcium dialyzability values than the raw kañiwa. On the other hand, calcium dialyzability was lower for the roasted and boiled quinoa (*Chenopodium quinoa*) than in the raw quinoa. According to the authors, cooking might increase the digestibility of the proteins with which calcium is bound, thus increasing the release of the mineral from any protein complexes. On the other hand, boiling might lead to an increase in mineral loss into the water.

#### Calcium salts and organic acids

Using an *in vitro* digestion/Caco-2 cell model, Etcheverry et al. (2005a) found no differences in calcium uptake results when human milk fortifiers (i.e., supplements containing protein, energy, minerals and an ample range of vitamins which are added to expressed human milk) were supplemented with three types of calcium salts: calcium glycerosphosphate gluconate, calcium phosphate, and calcium chloride.

Rao et al. (2007) used an *in vitro* solubility assay to measure calcium bioaccessibility from a commercial calcium-milk protein supplement. The results showed that the calcium present in this supplement was readily released by enzymatic digestion: with increasing pepsin concentration, more mineral was released from the supplement. This was probably a result of the proteolytic role that this enzyme has on the proteins present in this supplement, such as β-lactoglobulin, α-lactalbumin, and lactoferrin. Both β-lactoglobulin and α-lactalbumin have the ability to chelate/bind calcium. Thus, the proteolytic digestion of these proteins might liberate more calcium.

Organic acids might have an enhancing effect on calcium absorption (Pak et al., 1987). Perales et al. (2005) used Caco-2 cells to compare calcium uptake from infant formulas and from fruit juices containing milk and cereals (FMC). The calcium uptake was higher from the FMC samples than from the infant formulas, probably as a result of the presence of citric and malic acids in the juices. Shiowatana et al. (2006) also found an enhancing effect of citric acid on calcium absorption using a continuous flow dialysis system. The authors added organic acids to amaranth leaves and found that the enhancement on calcium dialyzability was most pronounced with the addition of citric acid followed by tartaric, malic, and ascorbic acids. The authors pointed out that the organic acids favorably affected calcium availability in spite of the likely presence of oxalate and phytate in the amaranth leaves. Bernardi et al. (2006) concluded that citric acid addition to a cookie formulation made with seeds of algarrobo (*Prosopis alba*), a leguminous tree, improved calcium dialyzability.

#### Recommended method

There are four methods for assessing calcium bioaccessibility and/or bioavailability: solubility, dialyzability, Caco-2 cell uptake, and transport. The Caco-2 cell model is a good model for predicting calcium bioavailability in humans (Cashman, 2003). The cells have features, including calbindin, vitamin D receptors, calcium transport channels, etc., that are essential for the study of vitamin D-mediated intestinal calcium absorption (Fleet et al., 2002). Furthermore, the *in vitro* digestion/Caco-2 transport method has been validated against human studies. When Mesías et al. (2009) compared two diets with different content of Maillard reaction products, the authors found no differences in calcium bioavailability results when studied in humans or in Caco-2 cells. The recommended method is therefore the *in vitro* digestion/Caco-2 uptake/transport method.

### Carotenoids

Carotenoids have received a lot of attention within the scientific community not only because some of them possess pro-vitamin A activity, meaning that they can be converted into retinoid forms, but because they can also act as antioxidants. There are over 600 carotenoids in nature, and they are responsible for the red, orange, and yellow colors of many fruits and vegetables. Beta-carotene, α-carotene, and β-cryptoxanthin (carotenoids with provitamin A activity), lycopene, lutein, and zeaxanthin (no pro-vitamin A activity) (Gropper et al., 2009) are the six most common dietary carotenoids. The consumption of carotenoids is inversely related to the incidence of cardiovascular diseases, cancer, cataracts, and age-related macular degeneration (Nagao, 2009), probably due to their antioxidant capabilities.

Food sources of carotenoids include plant foods such as carrots, sweet potatoes, tomatoes, kale, and spinach, to name a few. Carotenoid availability from plant foods is dependent on (1) factors that affect the food matrix in which the carotenoids are present and (2) the presence of certain dietary components (Yonekura and Nagao, 2007). In the food matrix, carotenoids are usually associated with proteins: carotenes and lycopene are found complexed to proteins in chromoplasts, whereas lutein is located in chloroplasts (Garrett et al., 2000). Food processing conditions (such as cooking, microwaving, and pasteurization) as well as the enzymatic processes during digestion that soften or break cell walls, disrupt the protein-carotenoid complexes, favoring carotenoid release, and bioavailability (Parker, 1996). Reduction in particle size (for instance through homogenization, grinding, or milling) will similarly favor carotenoid absorption. Certain food components will also affect carotenoid bioavailability. Once the carotenoid has been released from the food, it is incorporated into lipid droplets before entering the micelles, thus the presence of dietary fat will favor carotenoid absorption. On the other hand, the presence of soluble fiber as well as plant sterols and stanols, will negatively affect the absorption of carotenoids (Yonekura and Nagao, 2007).

#### Application of in vitro methods

A comprehensive literature search in PubMed revealed that there are basically three main *in vitro* methods to determine the bioaccessibility and/or the bioavailability of carotenoids from foods.

An *in vitro* solubility method for measuring carotenoids has been utilized for the bioaccessibility screening of multiple foods (Hedrén et al., 2002a,b; Mulokozi et al., 2004). The method consists of a digestion method that simulates the human digestive system, followed by an assessment via HPLC of the types and quantity of carotenoids released from the food. Following the intestinal digestion, the samples are centrifuged, and the aqueous portion is extracted with petroleum ether that is then evaporated. The residue, containing the released carotenoids, is dissolved in a mobile phase solvent (consisting of methanol, methyl-t-butyl ether, and water) and filtered through a 0.45 μm pore size cellulose membrane filter and subjected to reverse phase HPLC. This method has been used after minor modifications to study the effects of thermal processing (Lemmens et al., 2011) and particle size (Lemmens et al., 2010) on β-carotene bioaccessibility from carrots.

A modification of this method was introduced by Reboul et al. (2006). What is essentially different in this method is that following the *in vitro* digestion the samples are ultracentrifuged at very high speeds and the aqueous portion is collected and passed through a 0.22 μm filter, thereby obtaining micelles. Thus, the authors ultimately quantify the carotenoids present in micelles (i.e., micellarized carotenoids) as a measure of bioaccessibility. This method has been used to compare carotenoid bioaccessibility from durum wheat and egg pasta (Werner and Böhm, 2011) and from different varieties and species of citrus fruits (Dhuique-Mayer et al., 2007); and to assess the effect of thermal processing on lycopene bioaccessibility from tomato pulp (Colle et al., 2010), and others vegetables.

The study by Reboul et al. (2006) has been validated against human studies. The *in vivo* bioaccessibility results were obtained from a study published by Tyssandier et al. (2003). In this study, Tyssandier et al. (2003) measured the percentage of carotenoids recovered in the micellar phase (i.e., micellarized carotenoids) from human duodenum during digestion of a carotenoid rich meal. The meal contained sunflower oil, tomato puree (main source of lycopene), chopped spinach (main source of lutein), and carrot puree (main source of β-carotene). As reported by Reboul et al. (2006), the bioaccessibility values from the *in vivo* human results were in the same range as those measured after the *in vitro* digestion model, with the exception of spinach lutein bioaccessibility which was about fivefold higher in *in vitro* than in *in vivo* studies.

Results from the solubility assay agree with what is expected to occur *in vivo*. Cooking, which results in a more efficient release of carotenoids from the food matrix by softening cell structures so that digestive enzymes can work more efficiently, resulted in higher β-carotene release from carrots compared to the uncooked kind (Hedrén et al., 2002a). Homogenization, which represents a mechanical disruption of the tissue, resulted in a sevenfold and an almost fivefold improvement of β-carotene bioaccessibility from the raw and cooked carrot samples, respectively (Hedrén et al., 2002a). Reboul et al. (2006) similarly found that percent β-carotene bioaccessibility increased with the level of processing: 2.5–2.6% from canned or raw carrots, 4.4% from pureed carrots, and 14.1% from carrot juice.

Addition of cooking oil to the carrots increased the percent of β-carotene released from both the raw and cooked carrots, but the results were more significant with homogenized samples (Hedrén et al., 2002a). Addition of oil similarly resulted in higher bioaccessibility values from orange fleshed sweet potatoes (Bengtsson et al., 2009a). Cooking green leafy vegetables (leaves of amaranth (*Amaranthus* spp.), cowpea (*Vigna unguiculata*), sweet potato (*Ipomoea batatas*), pumpkin (*Cucurbita moschata*), and cassava (*Manihot esculenta*) in red palm oil instead of sunflower oil, resulted in 1.7–2.5 times as much bioaccessible β-carotene (Hedrén et al., 2002b).

Different cooking methods will affect in dissimilar manner the release of carotenoid from foods. Microwaved orange fleshed sweet potatoes resulted in lower β-carotene release either in the absence or presence of oil (without oil: 23.7%; with oil: 27.5%) than boiling or steaming (without oil: 38–40.7%, with oil: 45%) (Bengtsson et al., 2009a). The authors concluded that the short heating period for the microwaved samples was not sufficient to obtain an adequate breakdown of the sweet potato cell matrix and, subsequently, the release and transfer of β-carotene to the supernatant/micellar fraction was impaired.

It has to be kept in mind that carotenoids are susceptible to destruction by heat. Mulokozi et al. (2004) compared two cooking methods on carotenoid bioaccessibility and retention from diverse African vegetables: a traditional cooking method, which consisted of boiling samples for 20–30 min in the absence of oil, and a modified cooking method, which consisted of reduced boiling times, and thus a potential for reduced carotenoid destruction. Bioaccessibility of β-carotene from the traditional cooking method ranged from 5% to 26% and from 18% to 77% from the modified method. Losses of β-carotene were 14–51% from vegetables prepared via traditional methods and 6–34% when prepared with the modified method. Thus, while cooking will increase carotene release and bioaccessibility from the food matrix, it will also lead to a reduction in carotene concentration, due to destruction of the molecule.

Lycopene and β-carotene appear to be sensitive to digestive conditions. Déat et al. (2009) found there was a 25% loss of lycopene in a simulated gastrointestinal TIM model that measured bioaccessibility from a meal containing red tomatoes and sunflower oil. While lycopene appeared to be stable in the gastric and duodenal compartments, it was in part degraded in the terminal parts of the small intestine. Blanquet-Diot et al. (2009) also showed that lycopene, along with β-carotene, were sensitive to destruction. Recovery percentages of β-carotene were lower for a red tomato-containing meal than from a yellow tomato-containing meal (*P* < 0.05). On the other hand, zeaxanthin and lutein were stable during *in vitro* digestion.

Garrett et al. (1999) were basically the pioneers in the development of the Caco-2 method for carotenoid bioavailability. The method relies on an *in vitro* digestion followed by the addition of the aqueous, filtered portion of the digestate (which would be representative of micellarized carotenoids) to Caco-2 cells. The cells are then harvested in phosphate buffered saline, containing ethanol, and BHT (butylated hydroxytoluene, an antioxidant) and stored at −20°C. On the day of the carotenoid analysis, the carotenoids are extracted from cells with a series of acetone and/or hexane additions. The pooled hexane extract is then evaporated to dryness, reconstituted and analyzed by reverse-phase HPLC.

The method by Garrett et al. (1999) has been used to study the bioavailability of carotenoids from vegetables (Huo et al., 2007), spinach puree (Ferruzzi et al., 2001), and orange fleshed melons (Fleshman et al., 2011), among others. A very similar method was introduced by Liu et al. (2004). In this method the authors measured both bioaccessibility and bioavailability, but they did not ultracentrifuge nor did they filter the samples, thus they did not necessarily add micellarized carotenoids to the Caco-2. The authors found that cooking corn samples enhanced the amount of lutein (0.9 fold) and zeaxanthin (1.2-fold) taken up by the cells compared to the raw grain (Liu et al., 2004).

A concern with this bioavailability method has been the stability of the micellar carotenoids during the incubation time with Caco-2 cells. Some of these bioavailability studies have incubation times as long as 6 (Garrett et al., 2000) or 8 h (Liu et al., 2004). Oxidative reactions might modify and affect the quantity of carotenoids during their exposure to the Caco-2 cells, thus it is important to keep incubation time to a minimum while not affecting the sensitivity of this assay. Garrett et al. (2000) observed that the addition of 500 μmol/L α-tocopherol to the medium might confer protection against oxidation and thus improve the stability of carotenoids.

Interestingly, Biehler et al. (2011) found that the addition of calcium, iron, and zinc significantly reduced both micellarization and Caco-2 uptake of total carotenoids from a spinach meal by up to 55% (Ca) and 90% (Fe, Zn), respectively. The minerals, which had been added at concentrations ranging from 3.8 to 25 mM, can presumably interact with free fatty acids, forming insoluble soaps, and with bile acids, thus compromising carotenoid emulsification. Also, minerals might reduce the size of the micelles, resulting in a marked and significant decrease of carotenoids in the micelles. Bengtsson et al. (2009b) also found that iron inhibits β-carotene uptake by Caco-2 cells, and that an inverse relationship between the beta-carotene uptake and iron concentration in the test solution exists (*r*^2^ = 0.93, *P* < 0.05). With the addition of ferrous chloride (30 μM), the beta-carotene uptake was significantly reduced (*P* < 0.05), on average by 22%.

An extension to the above method involves transport studies in Caco-2 cells in which the cells are grown on Transwell inserts. Only a couple of transport studies have been conducted (O'Sullivan et al., 2008, 2010).

#### Recommended method and other comments

In all of the above methods, carotenoid bioaccessibility can be assessed; however, the Caco-2 method allows the measurement of both bioaccessibility and bioavailability. There are basically two *in vitro* solubility methods: one that measures soluble carotenoids and one that measures soluble micellarized carotenoids. In the first method there is always the possibility of overestimating the true bioaccessibility of carotenoids, because in the supernatant one is measuring carotenoids which are not micellarized as well as micellarized carotenoids. Micellarized carotenoids are obtained by measuring the fraction of the food carotenoid incorporated into the micelles (obtained from ultracentrifugation and filtration of the aqueous component through a 0.22 μM pore size membrane).

It is important to choose an *in vitro* method for carotenoid bioaccessibility that includes the extraction and measurement of carotenoids in micelles, the form in which the carotenoids will ultimately be absorbed by the intestinal cells. This is important for various reasons. First, there are compounds in foods that impair the transfer of carotenoids from the food matrix into the micelles, such as sucrose polyester, the structure in Olestra (Weststrate and van het Hof, 1995), fibers such as alginates, cellulose, and pectins (Yonekura and Nagao, 2009) plant sterols and stanols (Yonekura and Nagao, 2007) and divalent cations (Biehler et al., 2011). By the first solubility method, one could never assess this impairment in the carotenoid transfer from the food matrix to the micelle. Second, isomers of the same compound may incorporate into the micelle differently. For example, *cis* lycopene is more likely to be incorporated into micelles than *trans* lycopene, resulting in a higher bioavailability from the *cis* form than from the *trans* form. This might be as a result of a greater tendency for the *trans* isomer to form aggregates or due to its slightly lower solubility (Boileau et al., 1999; Failla et al., 2008). A higher micellarization was similarly reported for *cis* β-carotene than for *trans* β-carotene (Ferruzzi et al., 2006). This is of importance if different foods contain different amounts or ratios of *cis* and *trans* carotenoids. Third, different carotenoids might compete with each other at the level of entry into the micelle (van Het Hof et al., 2000) and different carotenoids might be incorporated into micelles differently. For instance, according to Garrett et al. (1999), the differential transfer of the carotenoids into micelles is dependent on their hydrophilicity. Carotenoids that have been released from the food matrix but are embedded in the very core of the fat droplet will not transfer to the micelle with the same ease as those carotenoids that are associated with the surface of the oil droplet. Thus, carotenoids like lutein are likely to be micellarized to a greater extent than α-carotene and β-carotene (O'Sullivan et al., 2010). Consequently, it is important to follow an *in vitro* digestion model that uses micelles to measure bioaccessibility.

An important question to ask is whether carotenoid bioaccessibility is a reliable predictor of bioavailability. According to O'Sullivan et al. (2010) and Garrett et al. (2000), this might indeed be the case: the amount of carotenoids present in the plant food and in their respective micelles will reflect the amount accumulated (a measure of uptake) and also secreted (a measure of transport) by Caco-2 cells. Thus, a measure of bioaccessibility might be sufficient as an estimation of how bioavailable the carotenoid is from the food in question.

When studying cellular carotenoid transport it is important to note that the presence of the cytosolic enzyme (β-C 15,15′-oxygenase) responsible for the cleavage of β-carotene into retinoids could affect the amount of carotenoids being released and consequently measured at the basolateral end. This is of no concern, however, when working with the parent line (HTB 37) of Caco-2 cells as this cell line does not produce the enzyme. However, in two clones of Caco-2 cells, PF11 and TC7, β-C 15,15′-oxygenase has been detected (During et al., 1998).

It is very difficult to compare results from different carotenoid *in vitro* bioavailability studies. As noted previously, one of the most important factors limiting the availability of carotenoids from foods is their release from the food matrix (Parker, 1996). Thus, not only will the species, cultivar, growth conditions, harvest method, storage conditions affect carotenoid levels in the food, the processing conditions will most certainly affect the bioaccessibility data. Added to this is the wide inter- and, even intra-, variations of different research laboratories in preparing the samples for *in vitro* digestion experiments, making the carotenoid bioavailability results very difficult, and almost impossible, to compare and make sense of.

Another problem one finds when reviewing the literature is the lack of homogeneity among different labs in presenting the data. For the most part, the results of carotenoid bioavailability are expressed as a percentage of the amount taken up by the cells, relative to the total amount of carotenoids in the micelles that are given to the cells. However, some authors express results in terms of the amount of absorbed carotenoids per cell protein. It would be advisable to present the data both as a percentage and as an absolute amount absorbed. A higher percent carotenoid uptake from one test meal versus another does not translate into a higher carotenoid amount taken up by the cells if the test meals have different carotenoid concentrations to begin with, or if the amount of carotenoids in the micelles is different.

### Folate

Folate is a very important vitamin for pregnant women and those of childbearing age due to its role in the prevention of neural tube defects, which can lead to congenital malformations like spina bifida and/or anencephaly where the brain has not developed. Worldwide, spina bifida and anencephaly are estimated to affect 225,000 children a year (Oakley, 2002). Folate also plays a role in the prevention of certain cancers (Rampersaud et al., 2002; Oaks et al., 2010; Williams et al., 2012), and of neurodegenerative and neuropsychiatric diseases, including Alzheimer's, dementia and depression (Kronenberg et al., 2009).

Food folate is present in orange juice, dark green leafy vegetables, dried beans and peas, asparagus, strawberries, and peanuts and exists as a pteroylglutamatyl form, which can have up to 9 glutamate residues (Gropper et al., 2009). The main pteroylglutamates in food are 5-methyl tetrahydrofolate (THF; 5-CH_3_-H_4_-folate) and 10-formyl THF (Gropper et al., 2009). The synthetic form of the vitamin, folic acid, is found in supplements as well as in fortified foods (Rampersaud et al., 2003) and exists as a monoglutamate. In the US, fortification of foods (such as bread, cereal, flour, pasta, and grain products) with folic acid was mandated by the Food and Drug Administration in 1998 in an attempt to prevent neural tube defects and other diseases.

Folate bioavailability is dependent on several factors including the intestinal deconjugation of polyglutamate folate, the stability of the vitamin before ingestion (i.e., during processing) and during digestion, the presence of compounds which might impact its stability, and the food matrix (McNulty and Pentieva, 2004). For folate to be absorbed, it has to be converted into its monoglutamate form by the brush border enzyme glutamate carboxypeptidase II (GCPII), also known as pteroylglutamate hydrolase, poly(glutamic acid) hydrolyse II, etc. Organic acids such as citric, malic, and phytic acid have been shown to inhibit this enzyme, thus reducing the bioavailability of polyglutamyl folates (Wei and Gregory, 1998). Furthermore, compounds in beans, banana, and spinach cause a moderate inhibition of the enzyme (35%), whereas tomato (46%) and orange juice (80%) cause a more drastic inhibition (Bhandari and Gregory, 1990).

Folate is a vitamin that can be unstable. Irradiation (Galán et al., 2010) and glycation, which is the binding of a protein or lipid molecule to a sugar molecule (Munyaka et al., 2010), have been shown to increase folate losses. Oxidation of folate, which results in inactive pterin and *p*-aminobenzoylglutamate compounds, is influenced by factors such as amount of oxygen present, temperature, pressure, pH, light, metal ions, and the duration of exposure to oxidants. Some compounds with antioxidant capabilities, such as ascorbic acid (AA), have an effect of stabilizing the vitamin, thereby increasing its bioaccessibility (Öhrvik et al., 2010).

The food matrix also plays a role. In a study by Castenmiller et al. (2000), the authors found that consumption of minced spinach, as opposed to whole leaf spinach, led to higher plasma folate levels in individuals. Similarly, microwaved chopped spinach led to higher plasma folate levels than microwaved whole spinach (van het Hof et al., 1999). Dietary fibers such as cellulose, lignin, pectin, sodium alginate, and wheat bran, appear not to affect folic acid bioavailability (Ristow et al., 1982).

#### The gastrointestinal model for measuring bioaccessibility of folate

Without a doubt, the method that has been used the most, in the past decade, to measure folate bioaccessibility is the dynamic gastrointestinal model (TIM) (Arkbåge et al., 2003; Verwei et al., 2003; Ohrvik and Witthöft, 2008; Öhrvik et al., 2010). It has been used to study both folate and folic acid bioaccessibility from foods like orange juice, breads, milk, and yogurt. Using this model, Verwei et al. (2003) found that folate binding proteins (FBPs) added to milk samples have different binding characteristics for folic acid and for 5-CH_3_-H_4_-folate. During gastric passage, a large fraction of folic acid remains bound to FBPs, whereas a large fraction of 5-CH_3_-H_4_-folate dissociates from the FBP, increasing the bioaccessibility of the vitamin. Fortification of milk with 5-CH_3_-H_4_-folate leads to higher folate bioaccessibility (~70%) than that fortified with folic acid (~60%). The authors attributed this difference to a lower binding affinity of FBP for 5-CH_3_-H_4_-folate compared with folic acid at the pH range of 5–7.4. A lower binding affinity could result in a higher release or dissociation of the folate compound from the folate-FBP complex during gastric passage and/or through the duodenum.

Arkbåge et al. (2003) also found a more pronounced inhibitory role of FBPs on folic acid than on folate (*P* < 0.05). In the absence of FPBs, folate bioaccessibility was 82% from yogurt fortified with folic acid and 5-CH_3_-H_4_-folate (Arkbåge et al., 2003). When FBPs were added, folic acid bioaccessibility decreased to 34% and 5-CH_3_-H_4_-folate bioaccessibility decreased to 54%. Interestingly, this study also found that FBPs were somewhat resistant to the digestive enzymes in the stomach and small intestine, and this resistance was dependent on the folate form present in yogurt. The FBP stability in yogurt fortified with folic acid (34%) was twice as high as the FBP stability in yogurt fortified with 5-CH_3_-H_4_-folate (17%). Thus, a relationship between the inhibitory effect of FBP on the bioaccessibility of folic acid and 5-CH_3_-H_4_-folate, and the FBP stability in folic acid and 5-CH_3_-H_4_-folate fortified yogurt appears to exist (Arkbåge et al., 2003).

While the TIM method allows the removal of digested material (along with the subsequent determination of folate) at any step of the digestion model, it only measures bioaccessibility, and not absorption. Absorption ultimately depends on the ability of the brush border enzyme glutamate carboxypeptidase II to deconjugate the polyglutamate forms of folate.

#### A method which incorporates the brush border enzyme

In 1998, Seyoum and Selhub incorporated a method in which the susceptibility of food folates to glutamate carboxypeptidase II was studied. In this method, the food was subjected to a peptic digestion at low pH and then incubated with a porcine jejunal brush border membrane extract which contained the hydrolase enzyme. The folate bioavailability index was assessed by comparing the concentration of the monoglutamyl folate in the experimental group to the total folate concentration in the control group as follows:

Folate bioavailability index=(M/T)×100

where M is the monoglutamyl folate concentration after treatment and T is the total folate concentration (5-CH_3_-H_4_-folate) in the control group.

The authors compared the folate bioavailability indices with the indices of bioavailability for the same foods (egg yolk, cow's liver, lettuce, lima beans, orange juice, cabbage, and baker's yeast) reported in human studies (Tamura and Stokstad, 1973; Babu and Srikantia, 1976). The results showed that the two sets of indices have a significant correlation (*P* = 0.068). Thus, this method measures the potential for food folates to be absorbed.

#### Recommended method

The main *in vitro* method which has been used to assess folate bioaccessibility is the dynamic TIM. This model mimics the human digestive system in a way that cannot be replicated by other *in vitro* systems. Effects like churning, peristaltic movements, flow of saliva, etc., are all replicated and controlled in the TIM. However, this model only measures bioaccessibility, and not absorption. Absorption of dietary folate ultimately depends on the ability of an intestinal enzyme located on the cell surface (called glutamate carboxypeptidase II) to deconjugate the polyglutamate form to the monoglutamate form. Thus, it is important not to rely solely on bioaccessibility results since absorption would ultimately depend on the deconjugation of folate and the effect that certain food components might have on the activity of glutamate carboxypeptidase II. Further studies which incorporate the susceptibility of food folates to the intestinal enzyme (Seyoum and Selhub, 1998) should be conducted.

### Iron

Iron deficiency is one of the leading risk factors for death worldwide, affecting an estimated two billion people (Zimmermann and Hurrell, 2007). The high prevalence of iron deficiency in the developing world has substantial health and economic costs, including poor pregnancy outcome, impaired school performance, and decreased productivity.

In humans, iron bioavailability is affected by dietary, luminal, and systemic factors. Dietary factors affect the solubility, the oxidation state of the mineral, or both, and include the iron absorption enhancers (AA, meat, poultry, and fish) and iron absorption inhibitors (phytate, egg yolk protein and egg yolk phosvitin, casein, conglycinin, albumin, and certain polyphenols). Calcium has been shown to impair both nonheme and heme iron absorption, which makes it different from the previous inhibitors that only affect nonheme iron absorption. The influence of vitamin A, carotenoids, nondigestible carbohydrates such as inulin (Hurrell and Egli, 2010), and oxalates on iron absorption remains unresolved. Luminal factors of the host include impaired hydrochloric acid and gastric secretions which could potentially reduce the digestive ability of the stomach and the solubility of the mineral as well as malabsorption syndromes that can affect iron absorption such as steatorrhea and tropical sprue (Beard et al., 1996). Systemic factors include iron status of the host, physiological state (e.g., pregnancy and obesity), genetics (e.g., hemochromatosis, thalassemias and related hemoglobinopathies), hormonal secretion (e.g., hepcidin), and chronic and acute infectious disease states.

Amongst all the nutrients in this review, the amount of information available for estimating iron bioavailability is certainly the most voluminous. Also, it is the micronutrient that has been validated the most against human studies (e.g., Au and Reddy, 2000; Yun et al., 2004). Below is a list of factors that have been studied in *in vitro* experiments.

#### Phytate

Several *in vitro* studies have been performed on the effect of phytate and dephytinization on iron bioavailability from plant-based foods. Afify et al. (2011) used an *in vitro* digestion/solubility assay to measure iron bioaccessibility from three white sorghum varieties (*Sorghum bicolor* L.). Iron solubility was 8.02–13.60% for the raw sorghum grains, 14.62–20.75% for the soaked grains, and 16.67–20.63% for the germinated grains. Soaking and germination are processes that activate the endogenous phytase present in the plant material. Soaking may also lead to a phytate reduction through water solubilization and subsequent leaching (from the food) of some phytic acid salts. Interestingly, after soaking and germination the iron *content* in the seed significantly decreased, which could be attributed to leaching of iron ions into the soaking medium (Afify et al., 2011).

Caco-2 iron uptake from infant cereals was improved after treatment with exogenous phytases (Frontela et al., 2009). Likewise, iron solubility of whole faba bean flours was significantly improved by phytate degradation (Luo et al., 2010). Total dephytinization of dehulled faba bean flour led to an increase in iron solubility, but dephytinization of hull flour had no effect on iron solubility. This is because the hull is rich in fiber and tannins, but has a low content of phytate compared to the dehulled faba bean. Phytate is more localized in the cotyledon of the bean. Treatment with endogenous phytases (achieved by incubating the samples at 55°C in the presence of acetate buffer) significantly decreased (*P* < 0.05) the total iron content of faba bean flour from 3.52 to 3.15 mg/100 g because of iron leaching into the medium. By contrast, when exogenous phytases were added, the total iron content was apparently less affected, probably because it was complexed with the added proteins (Luo et al., 2010).

Pynaert et al. (2006) compared processed vs. unprocessed complementary foods (CF) in Tanzania. The processed CF consisted of germinated, autoclaved and dried finger millet, kidney beans, roasted peanuts, and mango puree. The same ingredients in identical proportions were used for the unprocessed CF. Iron solubility was higher in the processed samples (19%) than in the unprocessed samples (5%) (*P* < 0.001). The *in vitro* solubility results, however, did not agree with a field trial in which no improvement in iron status could be demonstrated in children who were fed the processed food (Mamiro et al., 2004). The reduction in phytates by 34% and improvement in iron solubility to 19% due to processing might not have been enough to compensate for the rather low iron content of the complementary food.

Engle-Stone et al. (2005) studied iron bioavailability from an iron-phytic acid (PA) solution (1 Fe:20 PA molar ratios) with different amounts of AA added to achieve Fe: AA molar ratios of 1:0, 1, 5, 10, 20, 40, and 100. Caco-2 iron uptake from the 1:20 molar ratio of iron to phytic acid decreased Caco-2 cell ferritin formation by 91% in comparison to the control (i.e., Fe without PA). When AA was added (1:20:1 molar ratio of FeCl_3_:PA:AA) iron uptake increased by 180% relative to the control (i.e., Fe without PA or AA). Additional AA increased cell ferritin formation, but the effect was maximal at a 1:20:10 molar ratio of FeCl_3_:PA:AA. Clearly, the AA was able to partially reverse the effects of phytate inhibition under these conditions.

On the other hand, Beiseigel et al. (2007) found no differences in Caco-2 ferritin formation between two maize varieties, one of which contained more phytate (7% more) than the other. Adding AA to the two maize samples, significantly enhanced iron uptake from 2% to 7%. When Caco-2 values were compared to absorption values obtained from female participants who were fed the maize samples in the presence and absence of AA, the authors found that the Caco-2 model accurately predicted relative iron absorption from the maize meals (Beiseigel et al., 2007).

#### Iron salts

The bioaccessibility/bioavailability of different iron salts was also studied. Kapsokefalou et al. (2005) reported that iron dialyzability was higher in pasteurized milk samples fortified with iron pyrophosphate, ferrous lactate and ferrous bis-glycinate (*P* < 0.05) than with ferrous sulfate and ferrous gluconate. However, in commercial pasteurized and UHT milk products, there were no differences in dialyzable iron in products fortified with ferrous lactate or ferrous sulfate. Zhu et al. (2009) also found increased Caco-2 iron uptake from pure ferric pyrophosphate than from any pure iron compounds or chelates. Exposure of iron to pH 2 followed by adjustment to pH 7 markedly decreased FeSO_4_ bioavailability but had a smaller effect on bioavailabilities from ferric pyrosphosphate and sodium iron(III) ethylenediaminetetraacetate (NaFeEDTA), suggesting that these chelating agents minimize the effects of pH on iron bioavailability.

Kloots et al. (2004) found that the iron dialyzability was higher in chapatis (a typical Indian bread) prepared from whole-grain wheat flour fortified with NaFeEDTA or SunActive® Fe (ferric pyrophosphate) than those fortified with ferrous sulfate. Iron dialyzability from whole-grain wheat flour baked into chapatis was similar for all added iron sources (ferrous sulfate, ferrous lactate, ferrous fumarate, ferric pyrophosphate, carbonyl iron, electrolytic iron, Ferrochel® amino acid chelate, ferric amino acid chelate taste free [TF], and Lipofer™ which is a complex of ferric pyrophosphate, starch, and lecithin).

The effectiveness of disodium EDTA (Na_2_EDTA) on enhancing iron bioaccessibility was studied by Walter et al. (2003). Flour tortillas were fortified with different iron salts in the presence and absence of Na_2_EDTA. Iron dialyzability from flour tortillas fortified with reduced iron alone, reduced iron- Na_2_EDTA, ferrous fumarate Na_2_EDTA and native iron plus Na_2_EDTA were 8.8, 15.3, 10.2, and 18.2%, respectively. Native iron from corn-masa flour had a dialyzability of 1.4%, but upon addition of Na_2_EDTA, it increased to 18.2%. Like AA, Na_2_EDTA may combine with the iron fortificant, thereby enhancing dialyzability, with the advantage that it is stable during storage and processing. The authors conducted human iron absorption studies using the same flour tortillas used in the *in vitro* solubility studies. The human bioavailability results closely paralleled the ranks obtained in the dialyzability studies. The *in vitro* dialyzability and *in vivo* human absorption results were highly correlated (*r* = 0. 89, *P* < 0.001).

#### Protein

The variable effects of different proteins on iron bioaccessibility and/or bioavailability was assessed. Bosscher et al. (2001a) found that iron dialyzability was reduced by soluble dietary fiber. However, the inhibitory effect of soluble dietary fiber was more pronounced in casein than in whey-based formulas. Iron dialyzability from casein- and whey-based formulas supplemented with 0.42 g of locust-bean gum/100 mL were 0.32% and 1.45% (*P* < 0.05), respectively. Drago and Valencia (2004) similarly found a more pronounced inhibitory effect of casein than whey on iron dialyzability.

Hypoallergenic formulas (which were based on protein hydrolysates) resulted in the highest iron dialyzability values, followed by a preterm formula, the followup and soy, an adapted formula, and finally one without lactose (García et al., 1998). No differences, however, were observed in formulas having whey or casein as the main protein fraction.

The addition of milk to fortified fruit beverages containing either iron or iron and zinc had a positive effect on iron uptake by Caco-2 cells. There was a significant (*P* < 0.05) threefold increase in ferritin formation in samples with milk vs. no-milk added samples. Intact bovine milk proteins may maintain iron in a soluble form in the digestive tract, but inhibit its absorption unless the proteins are hydrolyzed. The increase in iron uptake could have been due to the effect of CPPs formed during gastrointestinal digestion (Cilla et al., 2008).

#### Polyphenols

In the presence of tannic acid (TA), Caco-2 iron uptake was significantly inhibited (98%) in comparison to the control (i.e., Fe without TA). An increase in cellular iron uptake was observed when AA was added at a molar ratio of 1:1:1000 Fe:TA:AA. However, the ferritin formation (i.e., iron uptake) at the 1:1:1000 Fe:TA:AA ratios was only half the ferritin observed for the control (i.e., Fe without TA or AA) (Engle-Stone et al., 2005).

Using the Caco-2 iron uptake assay, Miret et al. (2010) studied different food matrices (water, dough, powdered drink, and chocolate) containing one of the following iron forms: iron sulfate, hemoglobin, or sodium iron chlorophyllin, a water-soluble semisynthetic chlorophyll derivative where the magnesium in the porphyrin ring has been substituted by iron. Iron uptake from hemoglobin was not reduced by the dough but was significantly reduced by the powdered drink and chocolate (a source of polyphenols). This was interesting, since polyphenols are known to inhibit nonheme iron, not heme iron, absorption. According to the authors, polyphenols from wine and tea have been shown to increase pepsin activity, and this could influence the digestion of hemoglobin and the solubility of the released heme. Peptides derived from hemoglobin digestion are known to maintain heme solubility and to allow heme uptake. Extensive digestion of the peptides could decrease heme solubility and consequently, heme-iron bioavailability. Iron uptake from sodium iron chlorophyllin was significantly reduced by the dough and powdered drink but not by chocolate. However, the iron uptake of hemoglobin and sodium iron chlorophyllin was significantly higher than that of FeSO_4_.

A handful of studies have been conducted using the *in vitro* digestion/Caco-2 uptake model to compare white and red common bean (*Phaseolus vulgaris* L.) (Hu et al., 2006; Laparra et al., 2008; Tako et al., 2009; Tako and Glahn, 2010). All of them showed that Caco-2 iron uptake was lower from the red beans than from the white beans, probably due to the higher presence of polyphenolic compounds in the colored beans (Tako et al., 2009) that included flavonoids such as kaempferol and astragalin (Laparra et al., 2008). Animal trials were conducted with 1-week-old chicks (*Gallus gallus*) fed white beans and red beans with and without iron for 8 weeks. Following the 8 weeks, divalent metal transporter 1 (DMT1; iron-uptake-transporter), duodenal-cytochrome-B (Dcytb; iron reductase), and ferroportin (iron-exporter) expressions were higher (*P* < 0.05) in the intestines of the group fed red beans vs. other groups (i.e., groups fed red beans + Fe, white beans, or white beans + Fe). Higher expression of DMT1, Dcytb and ferroportin (as was seen in the red bean group) is indicative of a more iron deficient state (Tako and Glahn, 2010). Iron absorption from white beans was also higher in anemic piglets compared to red beans (14–16% vs. 9–10.5%, *P* < 0.05) (Tako et al., 2009).

The low cellular iron uptake results are supported by the lower dialyzability of Fe from colored beans (1.5-2.7%) than white beans (12.1-18.8%) (Laparra et al., 2008). Interestingly, there was no significant difference in iron uptake from red and black beans, in spite of differences in iron concentration. The MIB465 sample contained 49.7% more Fe (up to 30 μg g−1 of bean, dw) than DOR500, but both of the black bean (DOR500 and MIB465) genotypes exhibited no significant (*P* < 0.05) difference in Fe uptake (Laparra et al., 2008).

Beiseigel et al. (2007) found that following an *in vitro* digestion, Caco-2 cell uptake was higher from cooked great northern beans, which are white in color, than from cooked pinto beans, which are a mottled red color. Caco-2 ferritin values increased when the beans were mixed with orange juice, a source of AA. Human subjects were also fed the cooked beans with and without orange juice. When the *in vitro* data were compared to the *in vivo* data, the authors found that the Caco-2 cells inaccurately predicted lower iron bioavailability from pinto beans than from great northern beans, and a lesser enhancing effect of AA with pinto beans than with great northern beans.

#### Recommended method

Solubility, dialyzability, Caco-2 uptake and/or transport assays have all been used as iron bioaccessibility/bioavailability screening methods. It is important, however, to be cautious about solubility assays. A review by Miller and Berner (1989) concluded that discrepancies do exist between *in vitro* iron solubility and *in vivo* iron absorption results, especially when the effects of protein on iron bioavailability are being assessed. On the other hand, the authors stated that iron solubility appears to be a reliable indicator of AA effects on bioavailability. Dialyzability (Walter et al., 2003) and Caco-2 uptake studies (Au and Reddy, 2000; Yun et al., 2004) have been validated against human absorption results. However, a significant drawback to the dialyzability method is that when iron diffuses into the dialysis bag, during the intestinal digestion phase, a significant amount of the iron immediately becomes insoluble at the higher pH (van Campen and Glahn, 1999), which might significantly affect results. The *in vitro* digestion/Caco-2 uptake model is the recommended bioavailability method for iron, because it is an assay that can provide more information than bioaccessibility studies alone, such as the impact of food components on absorption rate and efficiency, and the possible competition amongst nutrients or between nutrients and food components for the same absorptive site.

### Magnesium

To the best of our knowledge, not much research has been conducted on magnesium bioaccessibility/bioavailability in spite of the fact that magnesium deficiency is a concern in the US. According to the 2005–2006 National Health and Nutrition Examination Survey (NHANES), 60% of all adult Americans do not meet the estimated average requirements (EAR) for this mineral (Moshfegh et al., 2009). A low magnesium status is associated with numerous pathological conditions, including atherosclerosis, hypertension, osteoporosis, diabetes mellitus, and some cancers (colon, breast), which has led the scientific community to conclude that magnesium deficiency is a greater nutritional problem than currently recognized (Nielsen, 2010). Fiber, protein, and phosphorus appear to affect magnesium bioavailability from foods (Institute of Medicine, 1997).

Using an *in vitro* digestion/solubility assay, Wróbel et al. (1999) found that magnesium solubility from Mexican maize tortillas was low (32.4%). This was probably a result of the high fiber/phytate content present in the maize tortillas. When Walter et al. (1998) supplemented diets containing maize, soybean meal, and corn starch with 0, 1, 2, 3, and 4% citric acid, they found an enhancing effect of citric acid on magnesium dialyzability. Magnesium dialyzability significantly increased with the addition of 1% and 2% citric acid. However, there were no differences in the percentage of dialyzed magnesium between the 2, 3 or 4% citric acid. Authors explained that the enhancing effect of citric acid on magnesium might be due to the high solubility of certain citrates formed in the digest after the addition of citric acid. Furthermore, there might be a ligand competition between the citrate and the phytate present in the meal.

#### Comments

None of the methods currently used to assess magnesium bioaccessibility/bioavailability have been validated against human absorption studies, and no method has been used extensively. Thus, there are insufficient data to make a recommendation on the most appropriate bioavailability/bioaccessibility method for this particular nutrient.

### Polyphenols

Of all the food components in this review, polyphenols comprise without a doubt the largest group of compounds. Polyphenols consist of several thousand compounds found in fruits, vegetables, and beverages. The polyphenols can be classified as flavonoids and non-flavonoids. Flavonoids consist of the flavonols, flavones, isoflavones, flavanones, anthocyanidins, and flavanols. The non-flavonoids comprise the phenolic acids (hydrobenzoic and hydroxycinnamic acids), lignans, and stillbenes (Table 2).

**Table 2:** **Polyphenols in foods**.

**Polyphenol**	**Compounds**	**Examples**	**Food sources**
Flavonoids	Flavonols	Kaempferol, quercetin, myricetin	Onions, kale, broccoli, apples, cherries, fennel, sorrel, berries, tea
	Flavones	Apigenin, luteolin, diosmetin	Parsley, thyme, celery, sweet red pepper
	Isoflavones	Daidzein, genistein	Soya bean, legumes
	Flavanones	Naringenin, eriodictyol, hesperidin	Citrus fruits, prunes
	Anthocyanidins	Pelargonidin, cyanidin, delphinidin, petunidin, malvidin	Cherries, grapes
	Flavanols	Catechins, gallocatechin	Tea, apple, cocoa
Phenolic acids	Hydroxybenzoic acid	Protocatechuic acid, gallic acid, p-hydroxybenzoic acid	Blackberry, raspberry, black currant, strawberry
	Hydroxycinnamic acids	Coumaric acid, caffeic acid, ferulic acid, synaptic acid, chlorogenic acid	Blueberry, kiwi, cherry, aubergine, apple, pear, chicory, artichoke, potato, corn flour, cider, coffee
Lignans		Secoisolariciresinol	Linseed, lentils, garlic, asparagus, carrots, pears, prunes
Stillbenes		Resveratrol	Grapes, pomegranate, groundnut

Adapted from Ross and Kasum (2002); Manach et al. (2004); Singh et al. (2008).

Polyphenols, unlike the other food components in this review, are not considered nutrients since they are not essential in our diet, in spite of the many health benefits they possess. Polyphenols have been associated with the prevention of cardiovascular heart disease, cancers, neurodegenerative diseases, and gastrointestinal disorders (González-Gallego et al., 2010).

Polyphenolic concentration in fruits and vegetables is dependent on many factors. In cherries, for example, the anthocyanidin and phenolic concentration is dependent on the cultivar, maturity, geographic location, and environmental factors such as light, temperature, and various stresses (Fazzari et al., 2008). Other factors that can affect polyphenolic concentration include soil type, rainfall, fruit yield per tree, whether cultured in greenhouses or in fields, etc. (Manach et al., 2004). Storage will also affect polyphenolic concentration (resulting in acceptable organoleptic changes like in black tea and in undesirable characteristics like the browning of fruits) as well as culinary methods (peeling, cooking) and industrial food processes (Manach et al., 2004).

The bioavailability of polyphenols is dependent on the food matrix and whether they can be released following digestion (Anson et al., 2009). Food polyphenols are usually bound to a carbohydrate moiety, forming glycones; without the sugar moiety, the simple polyphenol structure is an aglycone. During gastrointestinal digestion, the polyphenol is detached from the sugar resulting in a more absorbable compound. The bioavailability of some polyphenols, like quercetin and hesperidin, are strongly affected by the type of attached sugar (Scholz and Williamson, 2007). The presence of protein in a food matrix has been shown to form a complex with procyanidins, reducing the bioaccessibility of the compound (Keogh et al., 2007). Ferulic acid, one of the most abundant polyphenols in wheat grain, has a low bioavailability due to the fact that most of the ferulic acid cannot be released from the food matrix (Anson et al., 2009).

When absorbed, polyphenols are subjected to processes like methylation, sulfation, and glucuronidation inside intestinal cells. Those that are not absorbed will reach the colon where the microflora will hydrolyze the glycosides into aglycones and convert them into aromatic acids such as hydroxyphenylacetic acids from flavonols, hydroxyphenylpropionic acids from flavones and flavanones, and phenylvalerolactones and hydroxyphenylpropionic acids from flavanols, to name a few (Manach et al., 2004; D'Archivio et al., 2007). Some absorption of the polyphenols and enzymatic products might occur in the large intestine.

#### Bioaccessibility studies

The main *in vitro* bioavailability method that has been used repeatedly to measure the bioaccessibility of polyphenols is *in vitro* solubility. This method has been used to test the bioaccessibility of various polyphenols in extra virgin olive oil (Dinnella et al., 2007), orange (Gil-Izquierdo et al., 2001, 2003) and pomegranate juices (Pérez-Vicente et al., 2002), broccoli (Vallejo et al., 2004), cocoa liquor (Ortega et al., 2009), and raspberries (McDougall et al., 2005), among other foods. Fazzari et al. (2008) studied the polyphenol bioaccessibility of cherries (*Prunus avium* L.) of different degree of maturity. The authors found that the percent polyphenol bioaccessibility was higher in immature cherries (i.e., picked 1 week early) than the mature or overmature (i.e., picked 1 week late) cherries. Because immature cherries had a lower concentration of polyphenols, the actual bioavailable amounts of these compounds were lower than for mature and overmature fruit (Fazzari et al., 2008).

McDougall et al. (2005) studied the bioaccessibility of polyphenols from raspberries (*Rubus idaeus* L., variety Glen Ample) in the presence of different food matrices. Results showed that co-digestion of raspberries with commonly combined foodstuffs such as bread, breakfast cereal, ice cream, and cooked minced beef gave different patterns. Phenol bioaccessibility was slightly decreased by co-digestion with ice cream and cereal, whereas bread had no effect and minced beef caused an increase. Anthocyanin bioaccessibility was either unaffected or increased by co-digestion with the foodstuffs. Thus, anthocyanins may bind to food matrices during digestion, protecting them from degradation and increasing their bioaccessibility (McDougall et al., 2005).

#### Bioavailability studies

Only one *in vitro* polyphenol bioavailability study using Caco-2 cells has been conducted. This study assessed the absorption of resveratrol from boiled and roasted peanuts (Chukwumah et al., 2011). Digests of roasted peanuts showed higher resveratrol transport as opposed to boiled peanuts, even though bioaccessibility results were higher for boiled than for roasted peanut, which supports the idea that a higher amount does not necessarily imply higher bioavailability.

It is important to note that Caco-2 cells are able to metabolize some polyphenols. Kern et al. (2003) found that after a 24 h exposure of hydroxycinnamates to differentiated Caco-2 cells, several metabolites were generated including ferullic acid-sulfate, synaptic acids-sulfate, *p*-coumaric acid-sulfate, and methyl ferulate-sulfate. Similarly, incubation in the presence of diferulates resulted in free acid metabolites. Furthermore, after a 2 h incubation, only 10% of the original methyl ferulate (a hydroxycinnamic acid) was present in the media, disappearing completely by 4 h of incubation. Yi et al. (2006) who added anthocyanins from blueberries to Caco-2 cells grown on Transwell membranes, suggested that anthocyanins can be degraded and demethylated during absorption and transport by the cells.

Caco-2 cells therefore have the capacity to carry out processes like glucuronidation, sulfation and methylation which are normal metabolic processes that polyphenols undergo both in the small intestine and in the liver. Thus, it appears that in order to assess uptake or transport in this human cell line, following the incubation it would be appropriate not only to measure the original polyphenol present but also any possible metabolite/degradation products that might have resulted from it. This is something that would be very challenging to do if one is not aware of all the possible metabolic and degradation products that might arise.

#### Comments

For polyphenols, there is not a substantial amount of evidence as to which method is the most appropriate for measuring bioaccessibility/bioavailability. In general, *in vitro* methods are somewhat limited for the assessment of polyphenol bioaccessibility/bioavailability due to the active participation of the colon in the digestion and absorption of these compounds. An exception would be certain gastrointestinal models, like TNO's TIM model, which allow the incorporation of colonic fermentation experiments. Certainly, the *in vitro* solubility method has been utilized more frequently over the past 10 years than the other methods, and is more economical. No method has been validated against human absorption studies. Thus, there are insufficient data to make a recommendation on the most appropriate bioavailability/bioaccessibility method for this particular phytochemical.

### Vitamin B_6_

Vitamin B_6_ comprises a group of six related compounds: pyridoxal (PL), pyridoxine (PN), pyridoxamine (PM), and their respective 5′-phosphates (PLP, PNP, and PMP) (Institute of Medicine, 1998). The major forms in animal tissues are PLP and PMP; plant-derived foods contain primarily PN and PNP, sometimes in the form of a glucoside (Institute of Medicine, 1998). Vitamin B_6_ plays a role in modulating the actions of steroid and other hormones, glycogen degradation and amino acid metabolism. Because of its central importance in amino acid metabolism, requirements and reference intakes for vitamin B_6_ are usually expressed per gram of protein intake (Bender, 1994). While overt vitamin B_6_ deficiency is not a frequent finding nowadays in medical practice, evidence suggests that insufficiency of this vitamin is rather widespread in a quite large portion of the American population, especially in the elderly and in individuals with an alcohol addiction (Friso et al., 2012).

The known factors that impair the bioavailability of vitamin B_6_ from various foods include reactions that occur during food processing, reaction products that are formed in the presence of amino acids, fiber type and content, and the presence of a vitamin B_6_ glucoside (Reynolds, 1988). Pyridoxine-5′-β-D-glucoside (PN-glucoside) is a major naturally occurring form of vitamin B_6_ in fruits, vegetables and cereal-grains. The bioavailability of PN-glucoside as a source of vitamin B_6_ depends primarily on the extent of *in vivo* enzymatic hydrolysis (Nakano et al., 1997).

Ekanayake and Nelson (1986) measured the vitamin B_6_ bioaccessibility of a synthetic meal composed of casein (vitamin-free), Alphacell (non-nutritive bulk), maize oil, dextrose, and waxy maize starch. They were fortified to 20, 60 and 100% of the US recommended daily allowance levels for vitamin B_6_. The same diet was fed to rats. Vitamin B_6_ bioaccessibility results determined by this method showed a good correlation with the rat bioassay.

#### Comments

Based on an extensive literature search, there are currently two methods for assessing vitamin B_6_ bioavailability and/or bioaccessibility: solubility and uptake by Caco-2 cells. No method has been validated against human absorption studies. Thus, there are insufficient data to make a recommendation on the most appropriate bioavailability/bioaccessibility method for this particular nutrient.

### Vitamin B_12_

Vitamin B_12_, also known as cobalamin, is a water soluble vitamin that belongs to a group of compounds called “corrinoids” because of their corrin nucleus. Food sources of vitamin B_12_ are animal products including meat, meat products, poultry, fish, shellfish, and eggs. Milk and milk products contain less of the vitamin (Gropper et al., 2009). Its functions include homocysteine regulations, which may help decrease heart disease risk, and red blood cell production.

In foods, vitamin B_12_ is bound to proteins. The release of the vitamin from the food proteins is achieved by the gastric action of pepsin and hydrochloric acid. Vitamin B_12_ released from foods is first bound to haptocorrin (a protein found in saliva and gastric juice) also referred to as cobalophilin (Watanabe, 2007) or R protein (Quadros, 2010). In the duodenum, the complex is disrupted thanks to the action of pancreatic proteases. IF (intrinsic factor), a glycoprotein released by the stomach cells is associated with vitamin B_12_. The IF-vitamin B_12_ complex is then absorbed intact by intestinal cells in the distal ileum (Watanabe, 2007) through a receptor called cubilin (Quadros, 2010).

Vitamin B_12_ deficiency is common in people of all ages who consume a low intake of animal-source foods, and no vitamin B_12_ supplements or fortified foods. Malabsorption caused by atrophic gastritis or *Helicobacter pylori* infection, pancreatic or intestinal pathology, and gastric acid-reducing medications are likely to contribute to a deficiency (Park and Johnson, 2006). While vitamin B_12_ deficiency is more prevalent in developing nations, it is also prevalent in wealthier countries, among vegans and the elderly (Allen, 2010). In the US and UK, approximately 6% of those aged 60 or over are vitamin B_12_ deficient (Allen, 2009). The prevalence of vitamin B_12_ deficiency increases with advanced age, mainly because atrophic gastritis decreases the production of the acid and digestive enzymes needed to cleave the protein-bound vitamin B_12_ (Park and Johnson, 2006).

#### In vitro method

Because bioavailability of dietary vitamin B_12_ is dependent on the complex production and release of proteins from the mouth and stomach (i.e., haptocorrins and intrinsic factor), it is no surprise that there are no *in vitro* methods for studying vitamin B_12_ bioavailability from foods. There is one *in vitro* method which measures bioaccessibility (Miyamoto et al., 2009). In this method, the authors studied the bioaccessibility of vitamin B_12_ from Korean purple lavers, an edible alga, which appears to contain more vitamin B_12_ than other edible algae. Bioaccessibility was based on a gastric and intestinal digestion of the sample. Following the intestinal digestion, the samples were centrifuged and the soluble fraction was applied to a Sephadex G-50 fine gel filtration column. The macromolecular and free B_12_ fractions were estimated with blue dextran and pure B_12_ by measuring absorbance at 600 and 551 nm, respectively. The results indicated that the dried purple laver could be well digested only under the pH 2.0 conditions, but not under the pH 4.0 and 7.0 conditions. Under the pH 2.0 and 4.0 conditions, about half of the B_12_ found in the dried purple laver was soluble. Release of B_12_ from the purple laver was significantly decreased under the pH 7.0 conditions, a pH that serves as a model for severe atrophic gastritis, which prevails in elderly people (Miyamoto et al., 2009).

#### Comments

Unlike other nutrients, bioaccessibility of vitamin B_12_ does not equal bioavailability due to the complex physiological process involved in B_12_ absorption. An extensive literature review, did not reveal any *in vitro* method for measuring vitamin B_12_ bioavailability from foods except for the one previously discussed. The recommended method is to conduct absorption studies (using either fecal excretion or body retention methods) in human subjects.

### Vitamin D

Vitamin D, which was first identified as a vitamin early in the twentieth century, is now recognized as a pro-hormone. A unique aspect of vitamin D as a nutrient is that it can be synthesized by the human body through the action of sunlight, a characteristic that has made it challenging to develop dietary reference intake values (Institute of Medicine. Food, and Nutrition Board., 2011).

Vitamin D, also known as calciferol, comprises a group of fat-soluble seco-sterols. The two major forms are vitamin D_2_ and vitamin D_3_. Vitamin D_2_ (ergocalciferol) is made by plants, whereas vitamin D_3_ (cholecalciferol) is synthesized in the skin of humans from 7-dehydrocholesterol and is also consumed in the diet via the intake of animal-based foods. Both vitamin D_3_ and vitamin D_2_ are synthesized commercially and found in dietary supplements or fortified foods (Institute of Medicine. Food, and Nutrition Board., 2011). Circulating vitamin D_3_ is metabolized in the liver, by the enzyme vitamin D-25-hydroxylase, to 25(OH)D_3_, which is not biologically active. Activation requires its conversion to 1,25(OH)_2_D_3_ in the kidney by the enzyme 25(OH)D-1α-hydroxylase. Production of 1,25(OH)_2_D_3_ is tightly regulated by a number of factors, the most important of which are serum phosphorus and PTH levels (Tsiaras and Weinstock, 2011). It is unclear whether vitamins D_2_ and D_3_ are metabolized in the same manner.

Like other fat soluble vitamins, vitamin D is most absorbable when lipids are present. Other compounds, like dietary sterols, might affect vitamin D_3_ absorption. Using a Caco-2 cell model, Goncalves et al. (2011) found that vitamin D_3_ uptake was negatively affected by the presence of cholesterol and phytosterols in the mixed micelles. Sterols decreased the efficiency of vitamin D_3_ uptake by Caco-2 cells in a dose-dependent manner. These data were strengthened by the fact that phytosterols also significantly decreased vitamin D_3_ uptake in mouse intestinal fragments. According to the authors, the presence of sterols in the mixed micelles might have led to a different micellar structure that was less efficiently absorbed. Another possibility is a competition for uptake via a common membrane transporter. NPC1L1 (a transporter) has been described as the main cholesterol and phytosterol transporter in the small intestine but has also been involved in cholecalciferol uptake (Goncalves et al., 2011).

#### Comments

The study by Goncalves et al. (2011) is not a “standard” method for measuring vitamin D_3_ bioavailability from foods. For starters, the study did not include an *in vitro* digestion of the samples (mostly because pure vitamin D_3_ was used, as opposed to vitamin D_3_ from foods), but that could easily be incorporated into the methodology. Bioavailability of vitamin D_3_, unlike the bioavailability of other nutrients, is not exclusively dependent on the release of the nutrient from the food matrix (a measure of bioaccessibility) or on the absorption of the vitamin by intestinal cells (a measure of bioavailability). Vitamin D_3_ bioavailability is also dependent on the metabolism of the vitamin which includes the conversion of vitamin D_3_ into 1,25(OH)_2_D_3_. Thus, there is insufficient data to make a recommendation.

### Vitamin E

Vitamin E includes eight naturally occurring fat-soluble nutrients: α-tocopherol, β-tocopherol, γ-tocopherol, δ-tocopherol, and the tocotrienols (α-, β-, γ- and δ-). Alpha-tocopherol has the highest biological activity and the highest molar concentration of lipid soluble antioxidant in man. The commercially available synthetic forms of vitamin E are comprised of approximately an equal mixture of eight stereoisomeric forms of α-tocopherol, either unesterified or usually as the ester of acetate, succinate, or nicotinate. Supplements can contain either the natural *RRR*- or the synthetic (*all rac*) α-tocopherol (Brigelius-Flohé and Traber, 1999). In addition to its role as a potent antioxidant, vitamin E is involved in physiological processes, ranging from immune function and control of inflammation to regulation of gene expression and cognitive performance (Dror and Allen, 2011).

A handful of *in vitro* studies have been conducted to assess vitamin E release from foods. A comparative study of commercial pastas made with and without eggs showed that vitamin E bioaccessibility from pure durum wheat pasta (on average, 70.0 ± 4.2%) was significantly higher than that from egg pasta (on average, 49.4 ± 5.1%). The bioaccessibility of α-tocopherol tended to be higher than that of γ-tocopherol and β-tocopherol. The bioaccessibility of β-tocotrienol was higher than that of α-tocotrienol (Werner and Böhm, 2011). Granado-Lorencio et al. (2009) studied vitamin C-fortified juices with and without milk and iron. Vitamin E bioaccessibility was higher in the presence of milk and iron. The amount of α-tocopherol transferred into the micellar phase was slightly higher (although not statistically significant) in the presence of milk and in the presence of milk and iron than when the fruit juice was tested alone. However, the *in vitro* and *in vivo* results were inconsistent because the juice with the apparently higher *in vitro* bioaccessibility (fruit juice + milk + iron) showed the lowest serum response in thirty young women. Reboul et al. (2006) studied the bioaccessibility of a meal composed of boiled potatoes, minced beef, and olive oil, along with different foods rich in vitamin E (wheat germ oil, sunflower oil, hazelnut, almonds, wheatgerm, lettuce, Camembert cheese, apples, carrot, white wheat bread, fresh bananas, and cow's milk). They found that vitamin E bioaccessibility was extremely variable, ranging from 0.47% (from apple) to almost 100% (banana, white bread, and lettuce). With the exception of apple as source, α-tocopherol showed similar bioaccessibility (when sourced from almonds, wheat germ, cheese, and hazelnut) or higher bioaccessibility (when sourced from bananas, bread, lettuce, and milk) than γ-tocopherol.

Déat et al. (2009), who used the TIM coupled to Caco-2 cells, found that the percentages of α-tocopherol absorbed were significantly lower from a vitamin E-containing meal compared to the pure compound. The meal (as opposed to the pure compound) provides other components that may change the uptake behavior of vitamin E (other tocopherols, lecithins, etc.). Furthermore, other compounds present in the test meal might have competed with d-α-tocopherol for absorption through the SR-BI transporter (a transporter located on the intestinal cell surface).

#### Comments

Two methods have been used to measure vitamin E bioaccessibility: solubility and the TIM developed by TNO. The validation studies performed using solubility assays showed inconsistent results (Granado-Lorencio et al., 2009). The gastrointestinal model has not been used extensively, and has yet to be validated against human absorption data.

### Zinc

Zinc is a trace mineral with roles in cell growth and replication, bone formation, skin integrity, immune system function, and sexual maturation (Gropper et al., 2009). Its deficiency is very prevalent in the world, along with deficiencies in iron, vitamin A, iodine, and selenium (Etcheverry et al., 2005b). Populations with zinc deficiency are more likely to have infants born with neural tube defects (Dey et al., 2010), have higher incidences of infant and child mortality attributed to respiratory tract pneumonia (Barnett et al., 2010) and diarrhea (Luabeya et al., 2007), and exhibit a high incidence of child stunting (Umeta et al., 2003).

The bioavailability of zinc from foods is dependent on the presence of dietary components in the intestinal lumen. For example, iron inhibits zinc only when consumed in the form of supplements, in the absence of food, and when the iron to zinc molar ratio is 25:1 (Sandström et al., 1985). Below this ratio, the inhibitory effect is insignificant. Phytate and nucleic acids (all phosphorus containing compounds) decrease zinc absorption, and calcium might have a potential inhibitory effect on zinc but it appears that its role is evident only when phytate is present in the food (Davies and Olpin, 1979). High amounts of calcium may exacerbate the inhibitory effect of phytate on zinc absorption by forming a calcium–zinc–phytate complex in the intestine that is even less soluble than phytate complexes formed by either ion alone. Zinc binds tenaciously to proteins at near neutral pH. Thus, the amount and type of protein in the diet are factors that affect zinc absorption. In general, animal proteins, like beef, eggs, and cheese, have been shown to have a positive effect on zinc absorption, but such is not the case for casein. On the other hand, CPPs may affect zinc absorption in a manner different from casein (Lönnerdal, 2000).

The *in vitro* method that has been used the most for zinc is without a doubt the dialyzability method. Both solubility and dialyzability methods aim to estimate bioaccessibility, or the fraction of the mineral available for absorption. A report by Hunt et al. (1987), stated that neither the soluble zinc nor the amount of zinc associated with low molecular weight fractions (i.e., dialyzable zinc) were useful at predicting zinc availability *in vivo*. However, Hunt et al. (1987) used rat absorption data to reach these conclusions. Rats are among the animals that can produce phytase (Iqbal et al., 1994), an enzyme that digests inositol 6-phosphate, i.e., phytate. Contrary to Hunt's report, Chiplonkar et al. (1999) found that *in vivo* zinc dialyzability strongly correlated with *in vivo* human data. In this study, Chiplonkar et al. (1999) used meals (*n* = 23) from different published human studies and compared the human absorption data to their own zinc dialyzability results. The different meals contained rice, fruit, milk, legumes, cheese, peanut oil, sugar, etc. The results showed that the *in vitro* dialyzability method matched the human absorption data with a correlation coefficient of 0.925 (*P* < 0.001) (Chiplonkar et al., 1999; Hotz, 2005).

#### Effect of phytate on zinc

Luo et al. (2010) studied the zinc solubility from whole faba bean flour. Solubility was 31.6%, but it increased to 45.4% and 52.3% after the endogenous phytases were activated. Exogenous phytases, on the other hand, did not improve zinc bioaccessibility (35.4%). According to the authors, the added enzyme (which is a protein) might interact with zinc and prevent it from becoming soluble, in spite of the dephytinization. Both treatments (with or without exogenous phytases) reduced the total zinc content of faba bean flour, by 16% and 32% after a short and long incubation period, respectively, probably as a result of leaching into the medium.

Lestienne et al. (2005) evaluated the zinc solubility of whole pearl millet flour. Nondephytinized samples had a zinc solubility of 14.2%. After phytate degradation by endogenous phytases, zinc availability was increased to 23.8% and 27.4% after incubation for 1 and 3.5 h, respectively. However, treatment with exogenous phytases did not improve zinc bioaccessibility which could be due to the added proteins (i.e., enzymes) interacting with the zinc in the millet flour and preventing its solubilization. As noted by the authors, a review by Matsui (2002) reported that zinc bioavailability was not increased by addition of exogenous phytases in a number of animal studies. The authors also stated that some of the hydrolysis products of IP6 (i.e., phytate with 6 phosphate groups), particularly IP5 and possibly IP4 and IP3 (phytate with 3 phosphate groups), participate in the inhibition of zinc availability. Regarding the content of zinc, there were no differences between the treatment with and without exogenous phytases. On the other hand, the longer incubation period with the endogenous phytases significantly reduced zinc content (*P* < 0.0001) because of gradual zinc leaching into the medium.

An experiment by Bosscher et al. (2001b) done with infant formulas indicated that phytate:Zn molar ratios > 1.5, or [phytate]×[Ca]/[Zn] molar ratios > 200, can negatively affect zinc dialyzability. According to the authors, diets from which Zn availability is low include those that contain high phytate, soyabean-protein products, or have a phytate:Zn molar ratio > 15. Because of the synergistic effects between phytate and high Ca on Zn absorption, the [phytate]×[Ca]/[Zn] molar ratio of the diet is also frequently used to express Zn bioavailability.

#### Effect of protein on zinc

Zinc dialyzability was the highest from hypoallergenic infant formula consisting of protein hydrolysates (García et al., 1998). The zinc dialysis percentages (2.2–6.1%) obtained from the soy-based formulas were quite low, probably due to the concentration of phytate. There were no differences in the percentage of dialyzable zinc in formulas having whey or casein as the main protein source. The highest zinc content corresponded to the soy-based formulas. Drago and Valencia (2004) found that zinc dialyzability was adversely affected by casein content in infant formulas; the lowest values were found in formulas with the highest casein-to-whey protein ratio. Binding of a large proportion of zinc to casein may result in the entrapment of zinc in casein curds, which may be incompletely digested in the small intestine, thus rendering a significant proportion of zinc unavailable for absorption. Bosscher et al. (2001a) found that zinc dialyzability was inhibited by soluble dietary fiber. However, the inhibitory effect of soluble fiber on zinc dialyzability was more pronounced in casein than in whey-based formulas. Zinc dialyzability from casein- and whey-based formulas supplemented with 0.42 g of locust-bean gum/100 mL were 3.2 and 5.6% for zinc (*P* < 0.05), respectively.

#### Zinc salts

The bioavailability of zinc salts was also assessed via *in vitro* methods. Guillem et al. (2000) fortified milk and soy-based infant formulas with different salts. Dialyzability results from the milk formula were as follows (in decreasing order): oxide > gluconate = chloride = lactate = citrate > acetate, and from the soy-based infant formula were: gluconate > oxide > lactate = chloride = acetate > sulfate > citrate. According to the results obtained and without taking into account other factors that could also influence bioavailability, the choice compounds for zinc supplementation would be oxide and gluconate for milk-based products and gluconate and oxide for soy-based ones. According to the authors, dialyzability values have nothing to do with the water solubilities of the salts used. In fact, the higher dialyzability in milk-based formulas corresponded to one of the zinc compounds with the lowest water solubility (i.e., zinc oxide).

Finger millet was explored as a source of zinc fortification (Tripathi and Platel, 2010). Finger millet flour was fortified with either zinc oxide or zinc stearate. Zinc dialyzability from the fortified flour (with the different zinc salts) increased by 1.5–3 times relative to the unfortified flour. The bioaccessible zinc content in the unfortified finger millet flour was 0.18 mg/100 g, while that in the flours fortified with zinc oxide and zinc stearate was 0.25 and 0.49 mg/100 g, respectively. Thus, zinc stearate seemed to provide more bioaccessible zinc. Inclusion of EDTA along with the zinc salt significantly enhanced the bioaccessibility of zinc from the fortified flours, the increase being threefold. Inclusion of citric acid along with the zinc salt and EDTA during fortification did not have any additional beneficial effect on zinc bioaccessiblity. The [phytate]×[Ca]/[Zn] molar ratio in the finger millet flour was 329.1, which was brought down to 84.1 after fortification (Tripathi and Platel, 2010).

Using an *in vitro* digestion/Caco-2 cell model, Etcheverry et al. (2005a) found that addition of calcium glycerophosphate/gluconate (CaGPG) increased zinc uptake by Caco-2 cells from human milk fortifiers. Why CaGPG may have an enhancing effect on zinc is not known. Gluconate, present in CaGPG, may have an enhancing effect on zinc absorption.

#### Recommended method

There is a need for more studies that validate the *in vitro* methods for measuring zinc bioavailability. The Caco-2 model holds potential for studying zinc bioavailability. Zinc uptake in these cells has been characterized (Etcheverry and Grusak, in preparation), but certainly further studies are needed to assess how they can be used to study zinc bioavailability without the need to heat inactivate proteases (which might affect food nutrients and hence bioavailability). In the meantime, *in vitro* dialyzability assays might be the most appropriate method to study zinc bioaccessibility. It is the only method thus far that has been validated against human studies.

## Summary

Over the past 10 years, the number of published studies in carotenoids, iron, calcium and zinc bioaccessibility and/or bioavailability has been considerable compared to the other food components in this review. Only one vitamin D bioaccessibility study has been published within the past 10 years. There are, however, plenty of studies using Caco-2 cells to determine vitamin D-induced calcium transport (Fleet and Wood, 1999; Giuliano and Wood, 1991; Fleet et al., 2002) and to isolate and characterize the vitamin D receptors present in the basolateral end of the cells (Giuliano et al., 1991). Vitamin B_6_, vitamin B_12_ and magnesium have similarly not received a lot of attention, even though there is a growing number of individuals living in developed and developing countries who are deficient in vitamin B_12_ (Allen, 2009). The need to study these nutrients might not be as urgent as that for iron, vitamin A/carotenoids, zinc, and iodine which are the most prevalent micronutrient deficiencies in the world. Together with other vitamin and mineral deficiencies, including selenium, vitamin C and folate, they constitute the “hidden hunger”, a term that distinguishes this form of malnutrition from protein-energy malnutrition (PEM) (Etcheverry et al., 2005b). The lack of *in vitro* methods might have to do with the complexity of digestion/absorption involved with a particular vitamin or mineral, such as in the case of vitamin B_12_. No *in vitro* bioaccessibility/bioavailability studies were found for iodine, in spite of the fact that this is one of the most prevalent micronutrient deficiencies worldwide. While there are compounds in certain foods (e.g., broccoli, brussel sprouts, cauliflowers, etc.,) called “goitrogens” which affect iodine, they do so at the level of metabolism, affecting thyroid function and iodine assimilation.

It is noteworthy to point out that while conducting a literature search on *in vitro* bioavailability methods, more than a dozen studies focusing on the simultaneous determination of calcium, iron and zinc or iron and zinc bioavailability from foods were found (Bosscher et al., 2000, 2001a,c, 2002, 2003a,b; Jovaní et al., 2001; Sahuquillo et al., 2003; Etcheverry et al., 2004, 2005a; Lestienne et al., 2005; Frontela et al., 2009; Tako et al., 2009; Liang et al., 2010). The reason for these integrated approaches is probably based on the fact that these three minerals play an important role in adult and infant health and are susceptible to complex interactions.

For individual nutrients and food components, our review of the available literature has allowed us to draw several conclusions relevant to bioaccessibility and/or bioavailability. The recommended method for assessing calcium and iron bioavailability is the *in vitro* digestion/Caco-2 model. *In vitro* carotenoid bioaccessibility should be determined by a method that incorporates the extraction and measurement of carotenoids in micelles, the form in which these fat soluble components will ultimately be absorbed by the intestinal cells. *In vitro* dialyzability assays might be the most appropriate method to study zinc bioaccessibility, as it is the only method that has been validated against *in vivo* studies.

For certain nutrients and food components, namely magnesium, polyphenols, and vitamins D, B_6_, B_12_, and E, the existing data are not adequate to recommend which method is the most appropriate for the assessment of bioaccessibility/bioavailability. Researchers will need to assess the goals of their study to determine the approach that will provide the most relevant answers to their question of interest; or, they should consider using more than one of the approaches discussed in this review. The main *in vitro* method which has been used to assess folate bioaccessibility is the TIM. Further studies which incorporate the susceptibility of food folates to intestinal, enzymatic degradation (Seyoum and Selhub, 1998) should be carried out.

There is a need for more validation studies in which the *in vivo* results are compared to *in vitro* results. It is important to note that it is neither likely nor anticipated that any of the *in vitro* methods presented in this report will absolutely predict how much of a particular nutrient an adult human, child or infant will absorb and utilize. However, these *in vitro* methods can serve as useful preliminary screens that help us identify the most promising food matrix, processing conditions, staple crop, cultivar, growing conditions, etc., and their relative potential to impact nutrient bioavailability. Nonetheless, more research efforts should be applied to validating the existing *in vitro* methods, not only to determine if they are well correlated with human studies, but also to ascertain how to make meaningful improvements in the *in vitro* methods. In addition, researchers are urged to exercise caution in their use of the term bioavailability, and to be more explicit about which aspect of the bioavailability process they are measuring.

### Conflict of interest statement

The work of this paper was supported in part through funds from PepsiCo Inc. and from the US Department of Agriculture, Agricultural Research Service through Cooperative Agreement No. 58-6250-0-008. Any opinions, findings, conclusions, or recommendations expressed in this publication are those of the authors and do not necessarily reflect the view of the US Department of Agriculture or PepsiCo Inc., nor does mention of trade names, commercial products, or organizations imply endorsement by the US government or PepsiCo Inc.
